# Effects of Capsaicin on Biomimetic Membranes †

**DOI:** 10.3390/biomimetics4010017

**Published:** 2019-02-13

**Authors:** Neha Sharma, Huong T. T. Phan, Tsuyoshi Yoda, Naofumi Shimokawa, Mun’delanji C. Vestergaard, Masahiro Takagi

**Affiliations:** 1 School of Materials Science, Japan Advanced Institute of Science and Technology, 1-1 Asahidai, Nomi, Ishikawa 923-1292, Japan; s1440153@jaist.ac.jp (N.Sha.); huongptt@hnue.edu.vn (H.T.T.P.); tsuyoshi_yoda@aomori-itc.or.jp (T.Y.); nshimo@jaist.ac.jp (N.Shi.); takagi@jaist.ac.jp (M.T.); 2 Department of Food Science and Biotechnology, Faculty of Agriculture, Kagoshima University, 1-21-24, Korimoto, Kagoshima 890-0065, Japan

**Keywords:** capsaicin, liposomes, thermoresponsiveness, membrane fluctuation, membrane excess area, membrane fluidity, phase separation

## Abstract

Capsaicin is a natural compound that produces a warm sensation and is known for its remarkable medicinal properties. Understanding the interaction between capsaicin with lipid membranes is essential to clarify the molecular mechanisms behind its pharmacological and biological effects. In this study, we investigated the effect of capsaicin on thermoresponsiveness, fluidity, and phase separation of liposomal membranes. Liposomal membranes are a bioinspired technology that can be exploited to understand biological mechanisms. We have shown that by increasing thermo-induced membrane excess area, capsaicin promoted membrane fluctuation. The effect of capsaicin on membrane fluidity was dependent on lipid composition. Capsaicin increased fluidity of (1,2-dioleoyl-*sn*-glycero-3-phosphocholine (DOPC) membranes, while it rigidified DOPC and cholesterol-based liposomes. In addition, capsaicin tended to decrease phase separation of heterogeneous liposomes, inducing homogeneity. We imagine this lipid re-organization to be associated with the physiological warming sensation upon consumption of capsaicin. Since capsaicin has been reported to have biological properties such as antimicrobial and as antiplatelet, the results will help unravel these biological properties.

## 1. Introduction

Capsaicin (8-methyl-*N*-vanillyl-*trans*-6-nonenamide) is a chemosensory molecule derived from chili peppers of the *Capsicum* plant. It exhibits analgesic [1] and inflammatory properties [2], thus used to mitigate neurogenic pain and cure rheumatoid arthritis [3]. It has been reported that capsaicin can inhibit platelet aggregation [4], bacterial growth [5], and the expansion of prostate and lung cancer cells [6]. The compound also shows significant therapeutic and chemopreventive properties against certain mutagens and carcinogens [7]. In addition, capsaicin has been proposed to reduce adipose tissue and triglycerides [8], and to decrease appetite [9]. The exposure of nociceptor terminals to capsaicin leads to excitation of neurons and consequent perception of pain and local release of inflammatory mediators. With prolonged exposure, nociceptor terminals become insensitive to capsaicin, as well as to other noxious stimuli [10]. The long-term loss of responsiveness can be explained by the death of the nociceptor or destruction of its peripheral terminals following exposure to capsaicin [11]. 

The interaction of capsaicin with cell membranes is the crucial mechanism underlying its pharmacological and toxicological effects. It has been reported that capsaicin selectively binds to a protein known as transient receptor vanilloid 1 (TRPV1). The TRPV1 is a heat-activated calcium ion channel, which opens between 37 and 45 °C. When capsaicin binds to TRPV1, it causes the channel to open below 37 °C, thus capsaicin is linked to the sensation of heat [10]. However, some previous studies have suggested that a specific receptor is not the unique mechanism mediating the action of capsaicin. Capsaicin also interacts with the lipid bilayer of membranes [12,13], and this interaction is important to the effect of capsaicin on cells such as its ability to activate signals coming from a wide variety of receptors and membrane proteins, including the cystic fibrosis transmembrane conductance regulator (CFTR) [14] and the epidermal growth factor receptor (EGFR) [15]. 

Capsaicin is an amphiphilic molecule with a hydrophilic 4-hydroxy-3-methoxybenzyl-8-methylnon-6-enamide group and a hydrophobic 7-methyl-8-octene moiety. Thus, it is able to incorporate in the phospholipid bilayer and influence physicochemical properties of cell membranes such as membrane fluidity, ion permeability and phase separation [13,16,17]. Lundbaek et al. [18] have shown that capsaicin can regulate voltage-dependent sodium channels by altering the elasticity of the lipid bilayer. Using 1,6-diphenyl 1,3,5-hexatriene (DPH) fluorescent molecular probe and differential scanning calorimetric (DSC), Aranda et al. [13] have found a remarkable effect of capsaicin on the phase transition of 1,2-dipalmitoyl-*sn*-glycero-3-phosphatidylcholine (DPPC) liposomes, shifting the transition temperature to lower values. They have also reported that high concentrations of capsaicin decrease the fluidity of the membrane both below and above the transition temperature of the phospholipid [13]. Tsuchiya et al. [19] measured fluorescence polarization of *N*-phenyl-1-naphthylamine (PNA) and demonstrated that capsaicin has biphasic effects on liposomal membrane fluidity by fluidizing membranes at concentrations as low as 50–100 µM and rigidifying them at 100–500 µM. However, a recent research using DSC, X-ray diffraction, ^31^P nuclear magnetic resonance (NMR), and ^2^H NMR spectroscopy has shown that capsaicin increases fluidity and disorder of membrane models at all studied concentrations [20]. In addition, the manner in which capsaicin was introduced into the membrane lipids was different for these two studies. Capsaicin was introduced externally in Tsuchiya’s research, while Torrecillas et al. [20] incorporated the capsaicin during lipid vesicle preparation. These results suggest that the interaction between capsaicin and the membrane lipid bilayer is important but not yet well understood, and it is necessary to obtain more detailed information about the way in which this molecule modifies membrane physicochemical properties. 

In this study, we aimed to investigate the effect of capsaicin on cell-sized liposomes composed of 1,2-dioleoyl-*sn*-glycero-3-phosphocholine (DOPC), and cholesterol (Chol) with and without DPPC. DOPC and DPPC are unsaturated and saturated glycerophospholipids, respectively. They are the major constituent of lipids (40–60%) present in biological membranes, while Chol is very common in animal cell membranes, contributing to 30–40% of lipid composition [21]. The spontaneous self-assembly of DOPC and Chol generates homogeneous liposomes existing in liquid-disordered (Ld) phase, whereas liposomes composed of DOPC, DPPC and Chol are heterogeneous systems that tend to have a Ld phase coexisting with a solid-ordered (So) phase at low Chol fractions (0–10%) or liquid-ordered (Lo) phase at higher Chol levels [22]. These membrane systems, prepared as giant unilamellar vesicles (GUVs), are not only comparable to the actual cell membrane in size, lipid composition and bilayer structure, but also controllable [23]. Thus, they are great models for elucidating biomolecular interactions of interest, including oxysterols and polyphenols, and membrane lipid bilayers [24,25]. 

In order to assess how the location of capsaicin in the lipid bilayer and the capsaicin–membrane lipids interaction affect the physicochemical properties of the lipid bilayer, we synthesized liposomes with and without capsaicin. The temperature sensitivity of model membranes containing capsaicin was first measured through the direct observation of spatiotemporal membrane events captured in real-time. Then we examined changes in surface area to investigate the mechanism underlying thermo-induced morphological changes of membranes. In addition, we studied the effect of capsaicin on the fluidity and phase separation of the membranes. 

## 2. Materials and Methods

### 2.1. Materials

DOPC, DPPC and Chol were purchased from Avanti Polar Lipids, Inc. (Alabaster, AL, USA). Rhodamine B 1,2-dihexadeconyl-*sn*-glycero-3-phosphoethanolamine, triethylammonium salt (Rho-DPHE) (*λ*_ex_ = 560 nm, *λ*_em_ = 580 nm) and 6-dodecanoyl-2-dimethylamino naphthalene (laurdan, *λ*_ex_ = 430–455 nm, *λ*_em_ = 490–540 nm) were from Invitrogen (Carlsbad, CA, USA) and Funakoshi Co., Ltd. (Tokyo, Japan), respectively. Capsaicin was obtained from Sigma Aldrich Corporation (Tokyo, Japan). Chloroform and methanol were purchased from Kanto-Chemical Co., Inc. (Tokyo, Japan) and Nacalai Tesque, Inc. (Kyoto, Japan), respectively. All chemicals were of analytical grade and used as received. Deionized water was obtained using a Millipore Milli-Q (Millipore S.A.S., Molsheim, France), purification system, conductivity 18.2 MΩ cm^−1^. 

### 2.2. Preparation of Cell-Sized Liposomes

Four kinds of cell-sized liposomes, referred to as control (DOPC/Chol: 80/20 molar ratio), Cap 5% (DOPC/Chol/capsaicin: 76/19/05 molar ratio), Cap 10% (DOPC/Chol/capsaicin: 72/18/10 molar ratio), and Cap 20% (DOPC/Chol/capsaicin: 64/16/20 molar ratio) were prepared by the natural swelling method as described elsewhere [26,27]. To maintain stability in lipid vesicles, the control for the study was DOPC/Chol in the molar ratio of 80/20, otherwise addition of capsaicin would have made the liposomes unstable. The lipid mixtures dissolved in chloroform (2 mM) and capsaicin in methanol, were poured into glass tubes. For generalized polarization (GP) value determination, fluorescent probes Rho-DPHE (2 µL) and laurdan (6 µL) were added in the glass tube with lipids. The organic solvents were evaporated using a gentle stream of nitrogen to produce a thin film. The film was then dried under vacuum for 3 h and hydrated with Milli-Q water (200 µL) overnight at 37 °C. The final concentration of lipid was 0.2 mM.

### 2.3. Thermo-Induced Fluctuation of Lipid Vesicles

The liposomes were prepared using DOPC, Chol and capsaicin (at different concentrations) and incubated at 21.5 °C. At this temperature, the formed DOPC/Chol lipid vesicles exist in Ld phase and are stable. The liposome solution (5 μL) was transferred in a circular silicone well (0.1 mm) placed on a glass slide, and covered with a glass cover to prevent evaporation of solution. Observation of membrane dynamics was carried out using a phase-contrast microscope (Olympus BX50, Olympus Corporation, Tokyo, Japan) at temperatures ranging from 21.5 °C to 40 °C, increased at the rate of 1 °C/min using a thermo-controller (Tokai-Hit MATS-5550RA-BT, Tokai-Hit Co., Ltd., Fujinomiya, Japan) [25]. The temperature at which the lipid vesicles started to fluctuate was noted. The images were recorded on a hard-disk drive at 30 fps and processed using ImageJ software [28].

### 2.4. Effect of Temperature on Molecular Area of Monolayer Lipid Membranes

Changes in the molecular area of membranes caused by temperature change were measured using the Langmuir monolayer method. A Filgen LB-400 (Filgen, Inc., Aichi, Japan) instrument (Kuhn type) was used to measure the pressure–area (*p*–*A*) isotherms [29]. DOPC/Chol was dissolved in chloroform and capsaicin in methanol to a final concentration of 2 mM. This solution (5 μL) was gently added to the water–air interface of 100 mL of Milli-Q water at 20 °C, 24 °C and 28 °C. 

### 2.5. Multicomponent Phase Separation in Heterogeneous Membrane

Four kinds of heterogeneous membrane systems were prepared: DOPC/DPPC/Chol: 40/40/20 molar ratio), Cap 5% (DOPC/DPPC/Chol/capsaicin: 40/40/20/05 molar ratio), Cap 10% (DOPC/DPPC/Chol/capsaicin: 40/40/20/10 molar ratio) and Cap 20% (DOPC/DPPC/Chol/capsaicin: 40/40/20/20 molar ratio). Liposome solution was gently tapped and 5 µL was placed on a glass slide, used for microscopic observation. The liposomes were labeled with Rho-DPHE, which was localized with DOPC. Observation of domain localization was carried out using a fluorescence microscope (Olympus FV-1000, Olympus Corporation), within 1.5 min of addition of capsaicin. The observation temperature was maintained constant at 21.5 ± 0.5 °C. 

### 2.6. Measurement of Membrane Fluidity

The fluidity of membranes containing DOPC, Chol and capsaicin was measured using excitation GP of laurdan [30]. Laurdan fluorescent label was used at 0.02% (*v*/*v*) concentration. The final lipid concentration was 0.2 mM. Liposomes were observed at 473 nm and 559 nm (laurdan emission) using confocal laser scanning microscope (Olympus FV-1000 D, Olympus Corporation). Laurdan GP value was defined as GP = (*I*_(430–455)_ − *I*_(490–590)_)/(*I*_(430–455)_ + *I*_(490–590)_) in which *I*_(430–455)_ and *I*_(490–590)_ are average fluorescent intensities of laurdan detected at the ranges of 430–455 nm and 490–590 nm, respectively. 

### 2.7. Statistical Analysis

Image processing was performed using ImageJ software (downloaded from https://imagej.nih.gov/ij/download.html). The data are expressed as mean ± standard deviation (SD) of three independent experiments. Comparisons between different membranes were done using ANOVA followed by Bonferroni post comparison test. 

## 3. Results and Discussion

### 3.1. Thermosensitivity of Capsaicin-Containing Vesicles

**Figure 1:**
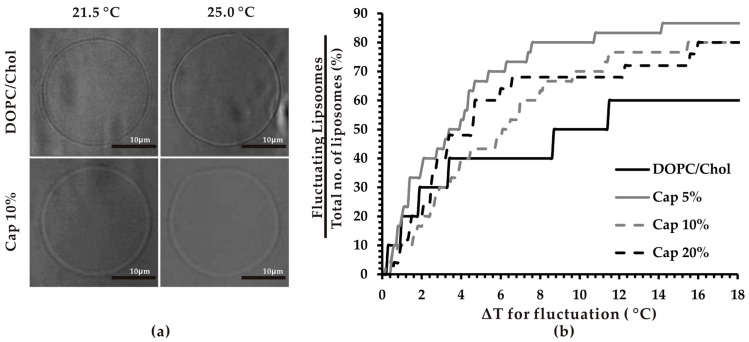
Thermosensitivity of capsaicin-containing vesicles. (**a**) Typical microscopic images of liposomes before and after fluctuation. DOPC/Chol liposomes (top) and DOPC/Chol/Cap (10% (*v*/*v*)) liposomes (bottom) at 21.5 °C and 25.0 °C as initial and final temperature, respectively. (**b**) Fluctuation profile of liposomes. Temperature was increased from 21.5 to 40 °C using a thermo-controller (tolerance ± 0.5 °C). The number of liposomes and the temperature at which the liposomes started to fluctuate was noted, expressed as a percentage of fluctuating liposomes over total number of liposomes, at the given temperature change (*n* = 30).

First, we investigated the effect of capsaicin on thermoresponsiveness of membranes by direct real-time observation of membrane fluctuation (Figure 1a). It has been reported that fluctuation is the initial step of most membrane dynamics. That is, visible lipid molecular re-structuring [23]. We calculated the percentage of fluctuating liposomes upon increase in temperature. The results showed that after ≈4 °C temperature increase, more capsaicin-containing liposomes underwent fluctuations compared to control vesicles. When the ∆*T* < 4 °C, the percentage of fluctuating capsaicin-containing liposomes, except for Cap 5%, was slightly smaller compared to that of liposomes without capsaicin. However, when ∆*T* > 4 °C, all capsaicin-containing lipid vesicles fluctuated significantly more than the control lipid vesicles (Figure 1b). This indicated that the presence of capsaicin in lipid bilayers increases the thermoresponsiveness of the bilayer. 

As discussed earlier, DOPC/Chol lipid vesicles are stable at 21.5 °C. In this study, temperature was increased steadily from 21.5 to 40 °C at a rate of 1 °C/min. This led to the thermo-induced movement of phospholipid acyl chains, observed as membrane fluctuation. Initial changes in temperature from 21.5 °C lead to fluctuations of a larger number of liposomes than the same at higher temperatures. This is not unexpected; it is known that membrane fluctuation is enhanced by an increase in the effective surface area (molecular packing) of membranes [26,27], reaching an equilibrium state at higher temperatures, where hydrophobic tension (the driving force for self-assembly of lipid molecules in water) counterbalances the inner pressure to move molecules further from each other when the temperature is increased. The ability to respond to temperature changes is one of the important properties of membranes, playing an essential role in maintaining fluidity and reducing the deleterious effects of temperature on membranes such as increased cellular permeability [31]. It might also contribute to the heat shock response of cells since the plasma membrane has been considered as the first responder to heat stress [32]. As far as we are aware, this is the first study demonstrating the effect of capsaicin on thermoresponsive fluctuation of the membrane lipid bilayer.

Noticeably, the increasing effect of capsaicin on thermoresponsiveness of membranes did not change linearly with concentration of capsaicin. Among studied capsaicin-containing systems, Cap 5% exhibited the highest fluctuation degree. The temperature difference needed for 50% of Cap 5% liposomes to start fluctuation was approximately 3.5 °C. When the capsaicin level in membranes was increased to 10%, thermo-induced fluctuation of membrane considerably decreased with 50% of liposome fluctuating when ∆*T* was around 6 °C. However, the highest concentration of capsaicin studied (20%) caused membranes to fluctuate more than at 10% concentration when the ∆*T* < 8 °C. When the ∆*T* > 8 °C, there was hardly any significant difference between Cap 20% and Cap 10% liposomes (Figure 1b).

### 3.2. Effect of Temperature on the Molecular Area of Monolayer Membranes

**Figure 2:**
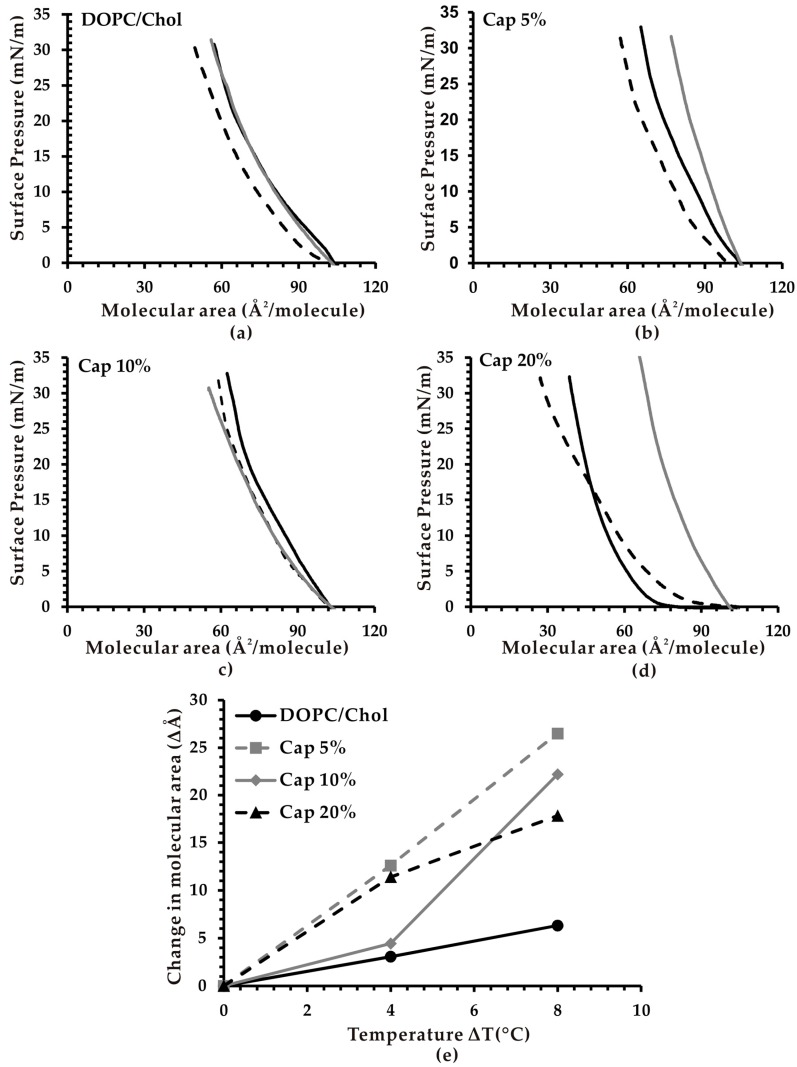
Typical thermoresponsive profile of lipid monolayers. Pressure–area curves of (**a**) DOPC/Chol, (**b**) Cap 5%, (**c**) Cap 10%, and (**d**) Cap 20% at 20 °C (black dashed line), 24 °C (black solid line) and 28 °C (gray solid line) (*n* = 10). (**e**) Graphical representation of the change in molecular area at 30 mN/m with respect to temperature.

It is reported that membrane fluctuation is caused by a decrease in volume (*V*) to area (*A*) ratio upon external physical stresses or surfactants [33]. These factors induce a membrane excess area or a reduce volume, thus decreasing the effective *V*/*A* ratio. Therefore, we hypothesize that the vesicle fluctuations observed in our study following the introduction of capsaicin leads to an increase in effective membrane area upon temperature changes. In order to confirm this, we measured changes in the molecular area of monolayers containing capsaicin using the Langmuir monolayer method. Figure 2a–d shows the *p*–*A* isotherms in the four studied monolayer systems, including DOPC/Chol, Cap 5%, Cap 10% and Cap 20% at different temperatures. We focused on the molecular area values at 30 mN/m surface pressure because it has been reported that normal lateral pressure in lipid vesicles is between 30 and 40 mN/m [29]. The changes in molecular area of all studied monolayer systems depending on the temperature at 30 mN/m was summarized (Figure 2e). 

The results revealed that at 20 °C and 30 mN/m surface pressure, capsaicin-containing monolayer membranes have smaller membrane area than the control. The membrane area of DOPC/Chol systems (control) was 60 Å^2^/molecule, while the values of Cap 5%, 10% and 20% were about 53, 47 and 40 Å^2^/molecule, respectively (Figure 2a–d). This could be explained by the localization of capsaicin in the lipid layers. Capsaicin molecules have a vanillyl group bearing a polar hydroxyl group at one end, which is connected via an acyl amide linkage to the lipophilic alkyl chain at the other end [13]. Previous studies have suggested that capsaicin occupies the region between the lipid–water interface and the double bond of the acyl chain in position *sn*-2 in the upper part of the lipid monolayer. The nine-carbon alkyl chain of capsaicin aligns itself principally with the main direction of the phospholipid hydrophobic chain. The hydroxyl groups locate near the interface of the lipid bilayer, allowing them to form hydrogen bonds with water and other hydrophilic interactions with the polar head of the phospholipids [13,20,34]. Due to the presence of only one polar OH group compared to a long hydrophobic side-chain and an aromatic ring, we proposed that the hydrophobic interaction of capsaicin is dominant and the molecule tends to place more inside the hydrophobic interior of the lipid layer. Thus, the partial substitution of DOPC and Chol with capsaicin in membranes leads to a smaller membrane area.

Interestingly, all capsaicin-containing monolayers exhibited a noticeably greater increase of membrane area upon increasing temperature in comparison with monolayers without capsaicin (Figure 2e). This result indicates that the inclusion of capsaicin in membranes accelerates the expansion of molecular surface area in response to temperature changes, leading to a higher membrane excess area, which in turn induces a larger number of lipid vesicles to fluctuate. This effect at increased temperature may involve the movement of capsaicin from within the lipid layers towards the membrane surface. A similar mechanism was previously reported in the case of oxysterols [25]. When ∆*T* < 4 °C, capsaicin may move slightly towards the surface, but it is still buried inside the lipid bilayer, resulting in a small membrane excess area, and insignificant fluctuation of the membrane. When ∆*T* > 4 °C, capsaicin may migrate even closer to the membrane surface, thereby generating a noticeable membrane excess area and fluctuation.

It is also interesting to note that the Cap 5% membrane system showed the highest increase in excess area with elevation in temperature. For the first 4 °C change, Cap 20% showed more increase in surface area than Cap 10%. However, it was reversed when ∆*T* = 8 °C (or subsequent 4 °C change). These observations correlated highly with the observed fluctuation behavior. 

### 3.3. Effect of Capsaicin on Membrane Fluidity

**Figure 3:**
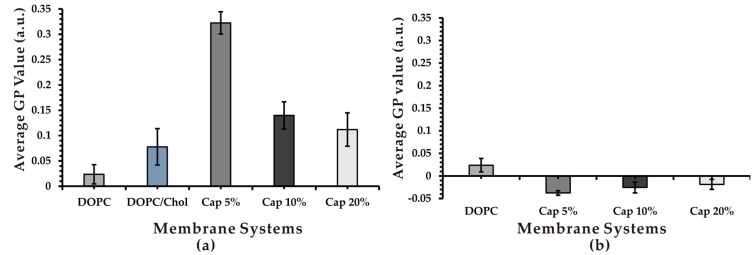
Generalized polarization values as membrane fluidity measured using a laurdan probe. (**a**) DOPC/Chol/Cap and (**b**) DOPC/Cap vesicles at different concentrations of capsaicin. a.u.: Arbitrary units.

Using a laurdan fluorescent molecular probe, we examined the effect of capsaicin on membrane fluidity, an important biophysical property of membranes. We used a laurdan fluorescence probe to investigate fluidity rather than a Langmuir monolayer because the laurdan fluorescence is the commonly used method for measuring bilayer fluidity while the latter is a fluidity measurement for lipid monolayers. The emission of laurdan fluorescence is sensitive to the polarity of its surrounding environment, which qualitatively corresponds to membrane fluidity. Membrane fluidity was measured using GP values. Generalized polarization values ranged from −1 to +1, implying the measurement of highest to lowest fluidity [30]. As shown in Figure 3a, the presence of capsaicin in DOPC/Chol membranes significantly decreased the fluidity of membranes compared to the control, as indicated by remarkably higher laurdan GP values. The rigidifying effect of capsaicin correlated inversely with its concentration. Cap 5% liposomes exhibited the highest GP value among all studied systems, indicating that they were the most rigid system. Liposomes with higher capsaicin concentrations (10% and 20%) were more fluidic than Cap 5% but still more rigid compared to the control. These results appear to contradict the previous findings of capsaicin’s fluidification effect on liposomal membranes reported by Torrecillas et al. [20]. The difference could be due to the difference in lipid composition of the liposome systems. In the previous study, the authors used a saturated phospholipid (DPPC) to prepare multilamellar vesicles, whereas liposomes used in our present work contain an unsaturated phospholipid and Chol in unilamellar vesicles. 

In order to investigate whether the presence of Chol, the main rigidifier of biological membranes [35], influences capsaicin’s effect on membrane fluidity, we measured the fluidity of liposomes containing only DOPC and capsaicin. Interestingly, the GP value of all DOPC/capsaicin membranes was significantly lower than that of DOPC systems, implying that the inclusion of capsaicin in DOPC membranes renders them more fluid (Figure 3b). However, the increasing concentration of capsaicin in DOPC membranes decreased its ability to fluidify membranes, as demonstrated by the higher GP value of Cap 10% and Cap 20% compared with Cap 5% (Figure 3b). The biphasic effects of capsaicin, fluidifying membranes at low concentrations and rigidifying them at high concentrations, have been reported previously in 1-palmitoyl-2-oleoyl-*sn*-glycero-3-phosphocholine (POPC) and DPPC liposomes [19]. Our results suggest that in addition to capsaicin’s concentration, lipid composition, in particular Chol, influences how capsaicin changes membrane fluidity. On the one hand, in membrane systems without Chol, capsaicin acts as a fluidifier. On the other hand, incorporation of Chol in membranes changes capsaicin from being a fruidifier to a rigidifier. 

Using the hypothesis of Gallová about the concentration-dependent differential interaction of heptacaine with membranes, Tsuchiya et al. [19] proposed that incorporation of capsaicin into lipid bilayers produces a free volume in the hydrophobic core of the bilayer. At low concentrations of capsaicin, acyl chains bend cooperatively and fill the free volume, thereby rendering membranes more fluid. However, at higher concentrations of capsaicin, the interdigitation of phospholipid acyl chains is formed to eliminate the free volume, leading to the rigidification of membranes [19]. Meanwhile, Torrecillas et al. [20] have reported that capsaicin is able to fluidize membranes by means of increasing the disorder of membrane lipid bilayers (at the concentrations used in this study). Capsaicin affects the hydrophilic part of the lipid molecules more than the hydrophobic region [20]. However, our findings suggest that addition of capsaicin to Chol-containing membrane systems tends to interact with Chol due to the ability to form hydrophobic interactions between its vanillyl moiety with hydrocarbon rings of the Chol molecule. The packing of capsaicin with Chol may inhibit capsaicin’s effect on the disorder of lipid bilayers and make the molecule act as a rigidifier. However, given the fact that a cell membrane tremendously varies in compositions, further studies are needed to clarify the mechanism behind the influence of lipid composition, particularly Chol, on the fluidizing behavior of capsaicin.

### 3.4. Phase Separation in Heterogeneous Membranes

Phase behavior is another important biophysical property of cell membranes. We therefore assessed the effect of capsaicin on properties of heterogeneous membranes composed of saturated phospholipid (DPPC) and unsaturated phospholipid (DOPC) with Chol. This ternary model system may form the Ld phase coexisting with So or Lo phases. The Ld and So phases are considered as liquid-crystalline and gel states of biological membranes, respectively, while Lo is composed of lipid raft-like domains [36]. In addition, few membranes exhibited no phase-separated boundaries (No domains). Heterogeneous liposomes were prepared with the three above components substituted by capsaicin at different concentrations (5%, 10%, and 20%). 

The results reveal that the introduction of capsaicin at 5% (Cap 5%) caused an increase of Lo domains, which may suggest incorporation of capsaicin within the DPPC/Chol-rich Lo domain. This behavior of capsaicin appeared to be opposite to that of the melittin molecule, which was reported to selectively insert into DOPC-rich domains of the same membrane systems [37]. The contradictory tendency of two molecules to locate in membranes can be explained by the difference in their molecular structure. Melittin is an amphiphilic α-helical peptide consisting of 26 amino acids with a large hydrophobic part (residues 1–21) and a short hydrophilic sequence (residues 22–26) [37]. Thus, it selectively targets and inserts into more fluid DOPC-rich domains, while in the DPPC/Chol-rich domain, all lipids are in gel state and the compact phase makes more difficult for melittin to act. In comparison, the capsaicin molecule is much smaller with a hydrophilic 4-hydroxy-3-methoxybenzyl-8-methylnon-6-enamide group and a hydrophobic 7-methyl-8-octene moiety [13]. The presence of a short acyl chain in the capsaicin molecule may render it able to fit in the compact phase of DPPC/Chol-rich domains. 

**Figure 4:**
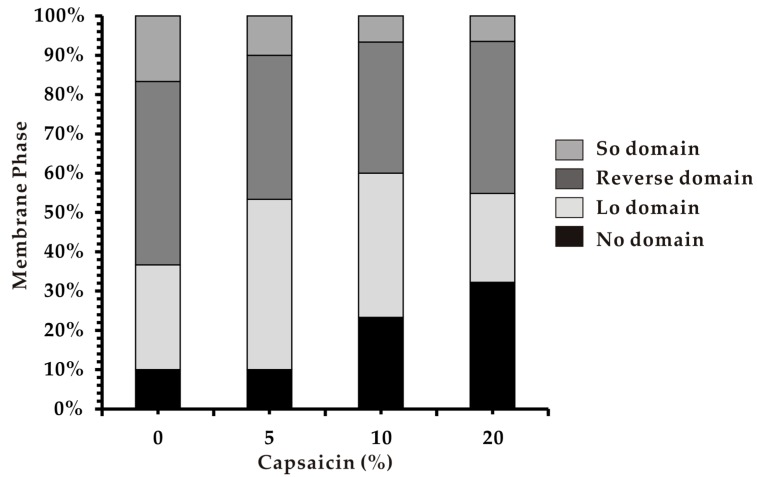
Phase separation in DOPC/DPPC/Chol/Cap systems. Lo: Liquid-ordered; No: No phase separation; So: Solid-ordered.

Afterwards, the number of No domains increased as membranes became homogeneous with increase in capsaicin concentration. The number of Ld domains also increased while the number of Lo domains significantly decreased (Figure 4). It was reported that heterogeneous model membranes become homogeneous in two cases when the ratio of Chol is high or with an increase in temperature [38]. Our results indicate that the effect of capsaicin on phase separation of membranes may fall into the latter aspect. That is, the elevation in capsaicin concentration, like the rise in temperature, renders membranes homogeneous. Although the presence of capsaicin was not externally introduced, the induction of a warming effect was observed. This property could be associated and could perhaps explain capsaicin’s activity as antiplatelet and antibacterial compound. A rise in body temperature is one of the first physiological defensive responses against microbial attack. 

## 4. Conclusions

In conclusion, we have demonstrated that capsaicin increased thermoresponsiveness of DOPC/Chol liposomal membranes. The inclusion of capsaicin resulted in greater thermo-induced membrane excess area, thereby prompting liposomes to fluctuate more upon increasing temperature. We have also found that capsaicin had an effect on membrane fluidity, and this was dependent on membrane lipid composition. Capsaicin tended to fluidize DOPC liposomes, while it acted as a rigidifier of Chol-containing liposomes. Of further interest was the effect on phase separation. Capsaicin was observed to induce homogeneity (one-phase) of liposomal membranes, from two-phase lipid domains. These findings will further the understanding of how capsaicin interacts with membrane lipids and the mechanism behind its warming sensation. Since capsaicin has been reported to be antimicrobial and as antiplatelet, the current findings on lipid structure re-organization thermally induced by the chilli compound could help to unravel its biological properties.
